# Real-Time Application of Artificial Intelligence for Automatic Detection of High-Grade Squamous Intraepithelial Lesions During High-Resolution Anoscopy

**DOI:** 10.3390/jcm15062268

**Published:** 2026-03-17

**Authors:** Luis Barroso, Miguel Martins, Maria João Almeida, Joana Mota, Francisco Mendes, Ahsan Javed, Amine Alam, Nadia Fathallah, Pedro Diaz Donoso, Dolores Caffarena, Luciana La Rosa, Thiago Manzione, Sidney Nadal, Simão Faria, Manuel Fortunato, João Ferreira, Guilherme Macedo, Vincent de Parades, Miguel Mascarenhas

**Affiliations:** 1 Section of Infectious Diseases, Department of Internal Medicine, Wake Forest University Health Sciences, Winston-Salem, NC 27157, USA; 2 Department of Gastroenterology, São João University Hospital, 4200-319 Porto, Portugal; 3 Hepatology Training Center, WGO Gastroenterology, 4200-427 Porto, Portugal; 4 Faculty of Medicine, University of Porto, 4099-002 Porto, Portugal; 5 Department of Surgery NHS, Royal Liverpool University Hospital, Liverpool L7 8XP, UK; 6 Department of Proctology, GH Paris Saint-Joseph, 75674 Paris, France; 7 Private Center of Surgery and Proctology, Buenos Aires C1060, Argentina; 8 Department of Surgery, Instituto de Infectologia Emílio Ribas, São Paulo 01246-900, Brazil; 9 Faculty of Engineering, University of Porto, 4099-002 Porto, Portugal

**Keywords:** high-resolution anoscopy, artificial intelligence, anal cancer screening, HSIL

## Abstract

**Background**: High-resolution anoscopy (HRA) is the gold standard for anal cancer screening, but its interpretation is challenging and operator-dependent. Artificial intelligence (AI) may increase diagnostic yield, but most studies have focused on differentiating low-grade and high-grade squamous intraepithelial lesions (LSIL and HSIL, respectively) in still frames, with no clinical application reported. **Methods**: We describe the first real-time use of deep learning, demonstrated in three patients undergoing HRA for anal cancer screening, at a high-volume American referral center. When an area suggestive of HSIL appeared, a YOLO-based object detection model generated a bounding box. **Results**: The model detected only histologically confirmed HSIL and did not activate when no lesions or only LSIL were present. **Conclusions**: This report suggests that real-time AI-enhanced HRA is feasible and may improve lesion detection and differentiation, potentially representing a significant step forward in this demanding field, although multicentric validation studies are still needed.

## 1. Introduction

The medical community is experiencing substantial advancements in artificial intelligence (AI), particularly in medical specialties that rely on image interpretation. AI models have been developed to address multiple challenges, ranging from lesion detection to the prediction of lesion histology [1].

In the anal cancer screening context, high-resolution anoscopy (HRA) is considered the gold standard for assessment of the anal canal, providing higher resolution and magnification for accurate diagnosis and treatment [2]. Detection and differentiation of HPV-induced lesions between low-grade and high-grade squamous intraepithelial lesions (LSIL and HSIL, respectively) is important as management differs. From a morphological perspective, LSIL often presents as faint or thin acetowhite staining, with a regular vascular pattern and preserved Lugol’s intake. HSIL presents as dense acetowhite, with an irregular vascular pattern and absent or partial intake of Lugol’s solution. From a clinical management perspective, LSIL often regresses and requires monitoring, whereas HSIL is precancerous and requires local treatment [3]. However, accurate detection and differentiation are challenging, given the variety of mucosal and vascular patterns, as well as the steep learning curve and high interobserver variability associated with HRA [4,5]. In the HRA case, the kappa coefficient between anoscopists can be as low as 0.076, highlighting the difficulty and the technical demands of the procedure [6].

These limitations are particularly concerning given the scale of the clinical need. Current estimates suggest that in the United States alone, nearly one million HRA would be required to screen at-risk populations, identifying more than 300,000 anal HSIL [7]. This demand is further amplified by the biologic nature of HPV-induced lesions, as HSIL tends to recur (approximately 50% within one year) [8], necessitating repeated procedures and increasing the projected annual volume to over 1.4 million HRA procedures.

Despite this growing clinical need, the number of trained HRA providers remains insufficient. This shortage is particularly explained by the technique’s steep learning curve, as limited experience has been associated with missed lesions [9]. According to IANS, a substantial number of HRA procedures is required to achieve a stable diagnostic accuracy plateau, as well as a minimum procedural volume to maintain this level of performance over time [10].

**Figure 1:**
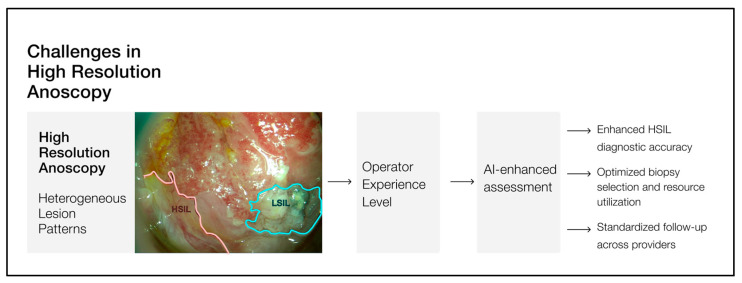
Main challenges in high-resolution anoscopy and how the development and application of artificial intelligence models can enhance its diagnostic performance.

The development and implementation of AI models in HRA have the potential to significantly enhance the diagnostic yield of the technique (Figure 1), not only by helping to bridge the gap between demand and high-quality HRA availability, but also by improving accuracy and procedural efficiency, even in expert settings. By reducing interobserver variability and increasing detection rates without increasing the false-positive rate, AI could have a beneficial clinical impact as an adjunct to a physician’s decision-making. To date, published studies have primarily focused on LSIL vs. HSIL differentiation in still frames, with no reports of real-time clinical application [11,12,13,14]. This case series presents the first reported use of a deep learning model for real-time assessment of the anal canal, in the context of anal cancer screening.

## 2. Methods

HRAs were conducted at a high-volume American center (Wake Forest University Hospital, West Salem, NC, USA) as part of anal cancer screening. Patient selection was entirely at the discretion of the institution. In addition to standard equipment, the HRA room was equipped with a computer connected to the HRA device, allowing real-time assessment with AI-enhanced predictions.

The model was trained on a multicentric dataset following FAIR principles, representing the latest iteration in the evolution of our group’s AI development regarding HRA, including a multicentric/multidevice dataset of 192.000 frames [15]. Annotations were performed by consensus of two physicians, taking into account histopathology assessment as the gold standard. A supervised object detection approach was employed with the backbone architecture being a pre-trained YOLOS vision transformer (ViT) [16] from Hugging Face. The final detection head of the network was adapted to match the number of classes in our dataset. The model was assembled with the PyTorch 2.9.1 framework. The AI algorithm was activated at the beginning of each procedure and, when an area suggestive of a lesion was detected, the model generated a bounding box locating the lesion(s). When an area suggestive of HSIL was detected within the image, the model generated a bounding box labeled “HSIL Lesion”. The physician could pause and resume the prediction process at any time during the examination.

## 3. Clinical Cases

### 3.1. Case 1

**Figure 2:**
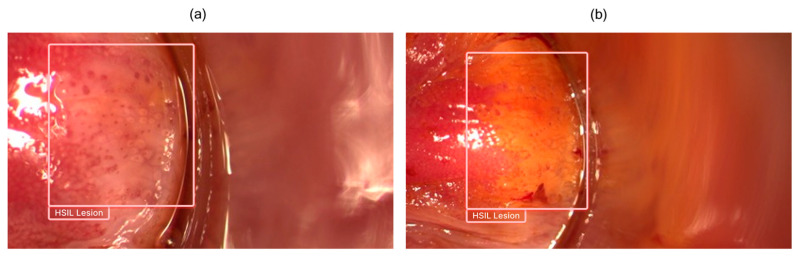
Real-time detection of HSIL. The AI model detected the lesion using a bounding box, delineating the suspected area. The detection remained stable under different staining methods: (**a**) acetic acid, (**b**) Lugol. Corresponding histopathology later confirmed the AI prediction.

A 53-year-old male patient with a medical history of congestive heart failure and diabetes mellitus was referred for HRA. A screening colonoscopy performed in July 2024 incidentally revealed anal LSIL. The patient was HIV-negative. A standard anoscopy conducted in October 2024 also demonstrated LSIL on biopsy. He was asymptomatic at presentation. During HRA, an acetowhite, Lugol-positive, verrucous, slightly elevated lesion was identified in the left lateral octant at the SCJ. Another acetowhite, Lugol-positive, flat lesion with a regular punctate pattern was noted in the right lateral octant at the SCJ. Both findings were compatible with LSIL. In addition, an acetowhite, Lugol-negative, flat lesion exhibiting a mosaic pattern, located in the anterior octant at the SCJ, was considered compatible with HSIL. During concurrent real-time assessment with the AI algorithm, only the anterior octant lesion was highlighted by a bounding box, indicating a prediction suggestive of HSIL (Figure 2). All three suspected lesions were biopsied. Histopathological analysis confirmed HSIL in the anterior octant, while the remaining lesions were LSIL. The patient was subsequently referred for HSIL treatment.

### 3.2. Case 2

A 38-year-old HIV-negative male patient presented for HRA. His past medical history included hypertension. Two months earlier, he had undergone a hemorrhoidectomy, during which an LSIL was incidentally identified on histopathological examination. He was asymptomatic at the time of evaluation. During HRA, cicatricial changes in the anal canal mucosa were noted, consistent with prior hemorrhoidectomy. In the right anterior octant at the SCJ, a flat, partially Lugol-positive lesion with regular micropapillae was observed, compatible with LSIL. Additionally, in the proximal perianal area of the left lateral octant, a distinct raised filiform lesion was noted, also consistent with LSIL. Concurrent real-time assessment by the AI algorithm did not generate any prediction suggestive of HSIL during the examination. All suspected lesions were biopsied, and histopathological analysis confirmed LSIL in all samples. The patient remains under clinical surveillance.

### 3.3. Case 3

A 40-year-old HIV-positive male presented for HRA. His HIV infection was well controlled. He had a history of HSIL detected during screening and had previously undergone electrocautery treatment in 2017. The patient was lost to follow-up but returned for evaluation and management of perianal condyloma. During HRA, no areas suggestive of HPV-induced lesions were identified. Concurrent real-time assessment with the AI algorithm did not generate any prediction suggestive of HSIL during the procedure. The patient remains under clinical surveillance. Table 1 summarizes lesion characteristics, real-time AI predictions, and corresponding histopathology diagnoses for all cases. In this summary table, “Yes” indicates that the AI algorithm generated a bounding-box prediction suggestive of HSIL, while “No” indicates the absence of an AI-generated HSIL alert.

Lesions identified during high-resolution anoscopy are shown with corresponding physician impressions, real-time AI predictions and final histopathologic diagnoses. HSIL, high-grade squamous intraepithelial lesion; LSIL, low-grade squamous intraepithelial lesion; N/A indicates not applicable since no biopsy was performed because no lesions were visualized; SCJ, squamocolumnar junction.

## 4. Discussion

As far as we know, these are the first reported cases worldwide of the clinical application of an AI model during HRA. While prior studies on AI model development for HRA have relied on retrospective analysis of still images, this deep learning model represents an incremental advancement that enables its integration into the live clinical workflow, as summarized in Table 2. It enabled successful real-time detection of HPV-induced precancerous lesions (HSIL), which were subsequently confirmed by histopathology. This approach may represent a significant step forward in improving anal cancer screening and its implementation, potentially enhancing diagnostic yield.

First, we should acknowledge that HRA, like any other medical procedure, should be performed by trained physicians, using the appropriate knowledge and equipment in accordance with the *leges artis*. The implementation of an AI model, as software as a medical device (SaMD), during HRA should enhance the physician’s work and never substitute their role or the role of tissue sampling in this context. Moreover, we cannot overlook the “black-box” nature of these algorithms, which prevents us from knowing exactly how the model generates its predictions. Explainable AI mechanisms, such as bounding boxes, are crucial for facilitating medical trustworthiness, but the final decision must always remain with the physician performing the procedure (“human in the loop”).

In HRA, where the multifocal nature of HPV carcinogenesis and the variability of superficial and vascular patterns of HPV-induced lesions make LSIL vs. HSIL detection and differentiation challenging, AI-enhanced HRA could increase diagnostic yield. On one hand, taking into consideration the steep learning curve and the high interobserver variability associated with the technique, this could increase the absolute number of detected HSIL per procedure. AI-enhanced HRA could therefore have a significant impact on reducing anal cancer incidence. On the other hand, considering that HPV-induced lesion differentiation is challenging even for expert physicians, the adjunct real-time guidance provided by AI could improve efficiency and support clinical decision-making regarding which lesions require treatment.

In this setting, the clinical value of an AI-assisted model relies not only on its ability to detect HSIL, but also on its behavior in the presence of non-HSIL lesions. In other words, when assessing the clinical importance of an AI model, it is important to consider not only its capacity to discriminate true positives but also true negatives. In this case, although the experience is limited, the model was activated in the presence of HSIL and did not activate in LSIL cases, reinforcing its potential clinical applicability.

Furthermore, when considering the mismatch between demand and availability, this type of AI-assisted model has the potential to bring a community setting closer to expert-level performance, where outcomes can be correlated with HRA volume and experience [9]. It may also contribute to the training and democratization of the technique across different clinical settings. Selection bias needs to be actively mitigated by training on datasets from multiple sites and devices, a process that has already been implemented in our group’s AI-enhanced HRA models, including the one currently applied in these cases. By enhancing generalizability, the multicenter/multidevice training approach may increase the technology readiness level (TRL) of such models, potentially improving the quality of anal cancer screening in high-risk populations.

Finally, one should consider that this clinical case report, while innovative, represents only a very limited experience with a small sample size and no external validation. Moreover, as these procedures were performed in a high-volume referral center, the results may not immediately reflect performance in a community or low-volume setting. True validation of the model must be conducted using a multicentric methodology with a larger number of cases. However, we believe this experience is worth sharing, given that this is the first report of real-time application of an AI model in such a demanding technique and serves as a proof of concept, demonstrating that it can operate effectively under real clinical conditions during live cases.

## 5. Conclusions

This is the first report of a real-time AI-enhanced HRA that suggests that the use of deep learning-based algorithms could generate accurate predictions and operate effectively not only in still frames but also during live procedures. This could reduce interobserver variability, shorten the technique’s learning curve and increase its adoption, particularly among less-experienced clinicians, while further enhancing expert diagnostic performance. Consequently, this could represent a significant step for anal cancer screening, enabling earlier and more effective treatments, although multicentric validation studies are still needed to confirm these findings.

## Figures and Tables

**Table 1:** Lesion characteristics, AI predictions and histopathology.

Case	Lesion Location	Physician Impression	AI Prediction (HSIL?)	Final Histopathology
1	Left lateral SCJ	LSIL	No	LSIL
	Right lateral SCJ	LSIL	No	LSIL
	Anterior SCJ	HSIL	Yes	HSIL
2	Right anterior SCJ	LSIL	No	LSIL
	Left lateral perianal proximal	LSIL	No	LSIL
3	No visible HPV-related lesions	Normal	No	N/A

**Table 2:** Evolution of AI models developed for high-resolution anoscopy. The current AI model was trained on a multicentric dataset (across 5 different centers and 5 different devices), using a total of 192.000 frames.

Study	Model Architecture	Training Data	Dataset Diversity	Clinical Key Strengths
Saraiva et al. 2022 [12]	Xception	5026 frames	1 center/1 device	Pilot study
Saraiva et al. 2024 [13]	ResNet	27,770 frames	1 center/1 device	Subanalysis per category
Saraiva et al. 2024 [14]	ResNet	57,882 frames	2 centers/2 devices	Contribute to interoperability
Mascarenhas et al. 2025 [11]	ResNet10	88,073 frames	4 centers/3 devices	Ubiquitous classification in anus and cervix
Martins et al. 2025 [17]	YOLO v11	192,000 frames	5 centers/5 devices	Explainable AI mechanism with bounding boxes
Current use in this case report	YOLO v11	192,000 frames	5 centers/5 devices	First real-time clinical case report

## Data Availability

Raw data were generated at the Faculty of Medicine of the University of Porto, Portugal. Derived data supporting the findings of this study are available from the corresponding author upon request.

## Abbreviations

The following abbreviations are used in this manuscript:

AI	Artificial Intelligence
HRA	High Resolution Anoscopy
HPV	Human Papillomavirus
LSIL	Low-grade squamous intraepithelial lesion
HSIL	High-grade squamous intraepithelial lesion
SCJ	Squamocolumnar junction
ViT	Vision Transformer
SaMD	Software as a Medical Device
N/A	Not applicable
